# Leveraging Single-Cell RNA-seq Data to Uncover the Association Between Cell Type and Chronic Liver Diseases

**DOI:** 10.3389/fgene.2021.637322

**Published:** 2021-03-08

**Authors:** Xiangyu Ye, Julong Wei, Ming Yue, Yan Wang, Hongbo Chen, Yongfeng Zhang, Yifan Wang, Meiling Zhang, Peng Huang, Rongbin Yu

**Affiliations:** ^1^Department of Epidemiology, Center for Global Health, School of Public Health, Nanjing Medical University, Nanjing, China; ^2^Department of Biostatistics, School of Public Health, University of Michigan, Ann Arbor, MI, United States; ^3^Department of Infectious Diseases, The First Affiliated Hospital of Nanjing Medical University, Nanjing, China; ^4^Department of Infectious Disease, Jurong Hospital Affiliated to Jiangsu University, Jurong, China

**Keywords:** chronic liver diseases, GWAS, scRNA-seq, integrated analysis, cell type

## Abstract

**Background:**

Components of liver microenvironment is complex, which makes it difficult to clarify pathogenesis of chronic liver diseases (CLD). Genome-wide association studies (GWASs) have greatly revealed the role of host genetic background in CLD pathogenesis and prognosis, while single-cell RNA sequencing (scRNA-seq) enables interrogation of the cellular diversity and function of liver tissue at unprecedented resolution. Here, we made integrative analysis on the GWAS and scRNA-seq data of CLD to uncover CLD-related cell types and provide clues for understanding on the pathogenesis.

**Methods:**

We downloaded three GWAS summary data and three scRNA-seq data on CLD. After defining the cell types for each scRNA-seq data, we used *RolyPoly* and *LDSC-cts* to integrate the GWAS and scRNA-seq. In addition, we analyzed one scRNA-seq data without association to CLD to validate the specificity of our findings.

**Results:**

After processing the scRNA-seq data, we obtain about 19,002–32,200 cells and identified 10–17 cell types. For the HCC analysis, we identified the association between B cell and HCC in two datasets. *RolyPoly* also identified the association, when we integrated the two scRNA-seq datasets. In addition, we also identified natural killer (NK) cell as HCC-associated cell type in one dataset. In specificity analysis, we identified no significant cell type associated with HCC. As for the cirrhosis analysis, we obtained no significant related cell type.

**Conclusion:**

In this integrative analysis, we identified B cell and NK cell as HCC-related cell type. More attention and verification should be paid to them in future research.

## Introduction

Chronic liver disease (CLD) is a public health topic of global concern. As estimated, about 844 million people worldwide are suffering from CLD and 2 million deaths each year (Asrani et al., 2019). Starting with diverse etiology-related chronic hepatitis, CLD might develop into cirrhosis and hepatocellular carcinoma after repetitive liver damage (Gadd et al., 2020). Environment risk factors associated with CLD are virus, diet, drug, and autoimmune (Marcellin and Kutala, 2018). With the development of molecular biology, the role of host genetic background in CLD has also gained wide attention (Anstee et al., 2020). Genome-wide association studies (GWASs) have contributed greatly to our understanding of the genetic roles in CLD pathogenesis and prognosis (Matsuura et al., 2017). A number of associated polymorphisms, including variants on *CDK14*, *SH2B3*, *CARD10*, *TLL1*, *PNPLA3*, and *HLA*, have been reported (De Boer et al., 2014; Sudlow et al., 2015; Matsuura et al., 2017; Nicoletti et al., 2017; Li et al., 2018; Ishigaki et al., 2020; Schwantes-An et al., 2020). Nevertheless, the current understanding of CLD is far from enough, and it is still of great significance to further clarify the pathological process of CLD and explore new treatment strategy for CLD patients (Marcellin and Kutala, 2018).

As the largest internal organ of the body, the liver consists of many cell types, including not only epithelial cells and some non-parenchymal cells (e.g., endothelial and mesenchymal cells) but also a variety of immune cells (MacParland et al., 2018; Aizarani et al., 2019; Ramachandran et al., 2019; Sharma et al., 2020). Different cell types vary greatly in abundance and function, leading to their completely distinct roles in the physiological and pathophysiological processes of liver diseases (Ramachandran et al., 2020). Single-cell genomics technologies are transforming our understanding on diseases like CLD, enabling interrogation of cellular diversity and function at unprecedented resolution, and adding a new dimension to traditional bulk transcriptomic techniques (Giladi and Amit, 2018). Single-cell RNA sequencing (scRNA-seq) has been used to feature the fundamental liver biology and the cellular mechanisms underpinning liver regeneration (Aizarani et al., 2019). It also has been used to uncover the pathophysiological changes of hepatic fibrosis and hepatocellular carcinoma, where the heterogeneity and changes of T cells (Zheng C. et al., 2017), macrophages (Ramachandran et al., 2019), and endothelial cells (Sharma et al., 2020) residing within the liver tissue may be critical in driving disease states.

Both GWAS and scRNA-seq have thrown light on the way to indepthly understand the pathogenesis of CLD and further laid a foundation for the development of precision treatment strategy (Saviano et al., 2020). Integrating GWAS summary data and scRNA-seq data to identify the cell types associated to CLD might provide new clues for understanding the pathogenesis of CLD (Calderon et al., 2017; Finucane et al., 2018; Hao et al., 2020). Here, we used *RolyPoly* and *LDSC-cts* to ensure the robustness and confidence of the result. Especially, we first processed the scRNA-seq data to derive averaged expression vector and differential expression gene (DEG) list of each cell type for *RolyPoly* and *LDSC-cts*, respectively. Then, we used the Ensembl database to obtain the position relationship between SNPs and gene (Yates et al., 2019). Finally, with GWAS data, scRNA-seq data and block annotation in place, as well as accounting for linkage disequilibrium (LD) of related population, we applied *RolyPoly* and *LDSC-cts* to identify and prioritize CLD-relevant cell types.

## Materials and Methods

### Genome-Wide Association Studies Data

The first category of summary statistics is Asian ancestry GWAS. The datasets are from the Biobank of Japan (BBJ)^1^ (Ishigaki et al., 2020). We focus on the CLD-related phenotype that contain allele information and variant ID and that contain effect size and its standard error. With the two criteria, we obtained two GWAS summary statistics: cirrhosis (*n* = 212,453, prevalence = 1.03%) and HCC (*n* = 197,611, prevalence = 0.94%). Here, cirrhosis and HCC in BBJ were adjusted for age, sex, and top five genotype PCs (Ishigaki et al., 2020). The details of the two GWAS data are provided in Supplementary Table 1. Based on Asian ancestry from the 1000 Genome Project (1000 GP), we filtered out variants with minor allele frequency (MAF) < 0.01 and Hardy–Weinberg equilibrium (HWE) < 10^–6^ (Auton et al., 2015). After these quality control (QC) steps, we finally obtained 7,246,475 and 7,246,543 SNPs from the two datasets.

The second category of GWAS summary statistics is from European ancestry. The dataset is from GeneATLAS website^2^ (Canela-Xandri et al., 2018). We focus on the CLD-related phenotype that contain allele information and variant ID and that contain effect size and standard error. With the two criteria, we obtain one GWAS summary statistics: cirrhosis (*n* = 452,264, prevalence = 1.99%). This cirrhosis GWAS data was adjusted for sex, array batch, UK Biobank Assessment Center, age, age2 (Sudlow et al., 2015), and the top 20 genotype PCs as computed by UK Biobank. The details of these data are also provided in Supplementary Table 1. Based on European ancestry from the 1000 Genome Project, we filtered out variants with MAF < 0.01 and HWE < 10^–6^ (Auton et al., 2015). After these QC steps, we finally obtained 7,636,847 SNPs from this dataset.

We treated the phase 3 of the 1000 Genome Project as the reference panel (Auton et al., 2015). Here, we collected 503 European individuals and 504 East Asian individuals with 81,271,745 SNPs. We used *PLINK* to calculate Pearson’s *r*^2^ values of pairwise SNPs for *RolyPoly* with the default 1 MB window size (Chang et al., 2015). In *LDSC-cts*, we set the window size to 1 centiMorgan to estimate LD scores (Finucane et al., 2018).

### Four Single-Cell Data

Considering the cirrhosis and HCC data acquired from GWAS, we searched the GEO database for related scRNA-seq data and obtained one data for liver cirrhosis and two for HCC, whose raw counts data are available (Barrett et al., 2012; Ramachandran et al., 2019; Losic et al., 2020). In addition, to verify the specificity of the outcomes, we also downloaded an idiopathic Parkinson’s disease (IPD) data. The details are provided in Supplementary Table 2. Following the original study, we performed QC and clustering for each scRNA-seq data. Note that scRNA-seq data usually have the potential to have its clusters continuously subdivided, but we just controlled the cell type number of each data within 10–20 depending on the features and quality of each data. The specific processing details of each data are as follows: After demultiplexing, aligning, and estimating cell-containing partitions and associated UMIs, a cirrhosis dataset (GSE136103) consisting of CD45 + and CD45-, blood and liver, healthy and cirrhosis, and human and mice samples were downloaded (Ramachandran et al., 2019). Here, we only chose nine human cirrhotic samples, including five CD45 + and four CD45- samples, for downstream analysis.

**FIGURE 1:**
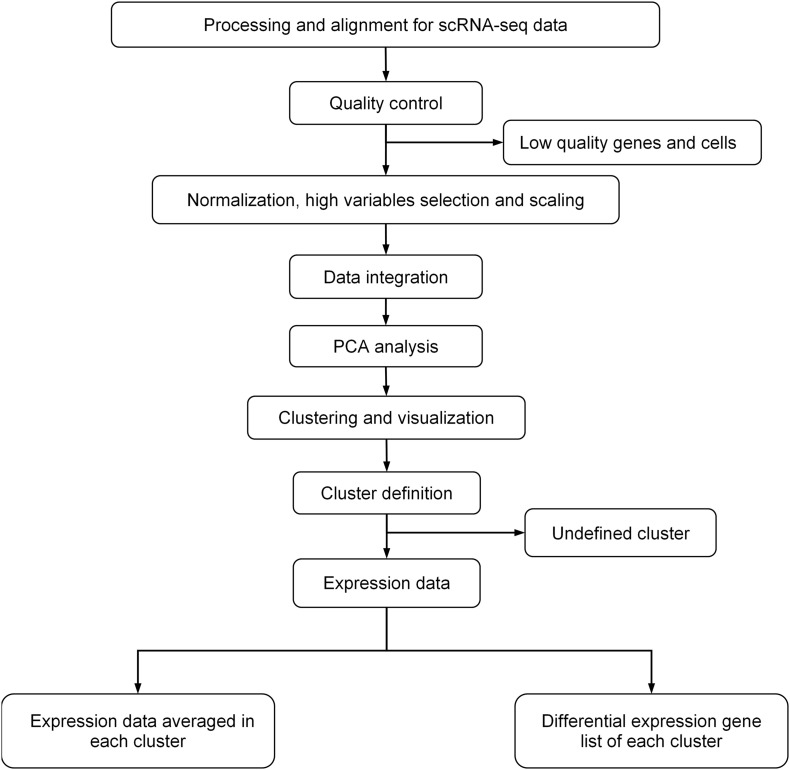
General procedure for scRNA-data processing.

For scRNA-seq data analysis, we first removed potential doublets, and then excluded the cells that expressed fewer than 300 genes or mitochondrial gene content >30% of the total UMI count (Ramachandran et al., 2019). We also excluded genes expressed in fewer than three cells. We followed the analysis flow in *Seurat* (Stuart et al., 2019): (1) used *SCTransform*, a new strategy to remove the influence of technical characteristics while preserving biological heterogeneity via regularized negative binomial regression, to normalize and scale scRNA-seq data (Hafemeister and Satija, 2019); (2) used default setting of *IntegrateData* to remove the batch effect (Butler et al., 2018); (3) performed unsupervised clustering and differential gene expression analyses on the integrated data; (4) used principal component analysis (PCA) for linear dimension reduction, and then used shared nearest neighbor (SNN) graph-based clustering, in which the graph was constructed using the top 30 principal components; and (5) used UMAP to visualize by the same number of principal components (PCs) as the associated clustering, with perplexity ranging from 30 to 300 according to the number of cells in the dataset or lineage. The details of data processing are shown in Figure 1.

**FIGURE 2:**
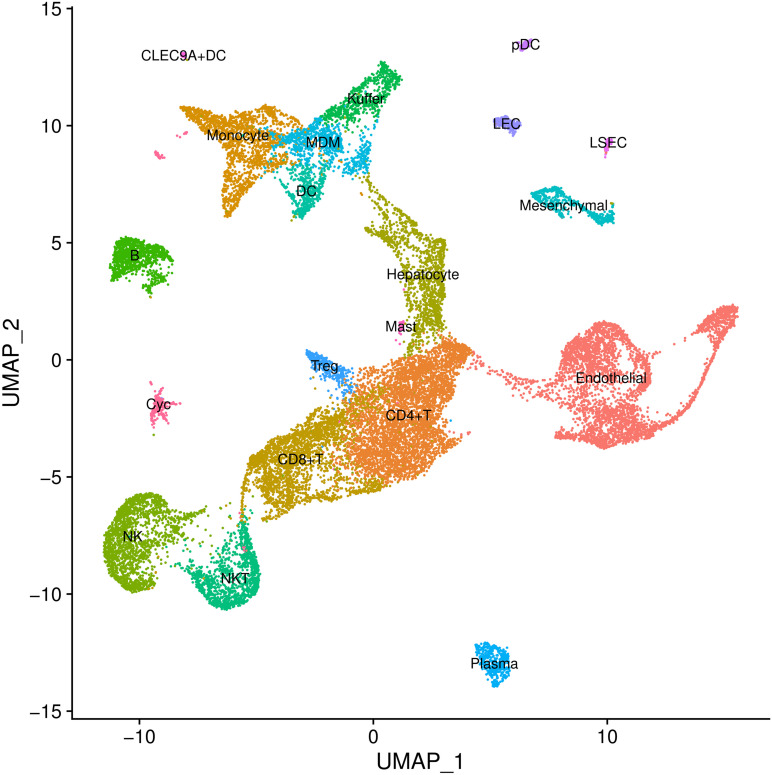
Cell types inferred from expression of marker gene signatures in GSE136103. NKT, natural killer T cells; Pdc, plasmacytoid dendritic cell; Treg, regulatory T cell; LEC, lymphatic endothelial cell; MDM, monocyte-derived macrophage; NK, natural killer cell; LSEC, liver sinusoids endothelial cell; DC, dendritic cell.

In cell type definition, we referred to marker genes that are widely recognized and those from the original research. We used *BuildClusterTree* to assess cluster similarity by constructing the phylogenetic tree (Stuart et al., 2019). Totally, we identified 20 clusters on 23,184 cells (Supplementary Table 2 and Figure 2). Marker genes used for cell type definition are shown in Supplementary Table 3.

The first HCC dataset (GSE149614) contains 21 primary tumor, portal vein tumor thrombus (PVTT), metastatic lymph node, and non-tumor liver samples from 10 HCC patients. We downloaded the raw count data, which have been processed and aligned by Cell Ranger, and chose only 10 primary tumor samples for downstream analysis (Zheng G.X.Y. et al., 2017). After processing and clustering, we totally identified 14 cell types on 30,983 cells in this dataset (Supplementary Table 2).

Another HCC dataset (GSE112271) contains three and four tumor samples coming from different regions of two different individuals, and we included all seven samples for downstream analysis. After data processing, we totally identified 13 clusters on 32,200 cells in this dataset (Losic et al., 2020; Supplementary Table 2).

We downloaded the processed and aligned IPD dataset (GSE157783), which contains samples from six control and five idiopathic Parkinson’s disease cases. We chose only five disease samples for downstream analysis and totally identified 12 clusters on 19,002 cells following our procedure (Supplementary Table 2).

### Defining the Specific Cell Types Associated With Cirrhosis and HCC

We used *RolyPoly* and *LDSC-cts* to define the specific cell types associated with cirrhosis and HCC (Calderon et al., 2017; Finucane et al., 2018). Based on polygenic model, *RolyPoly* treats the variance of each gene as the linear combination of each cell type and estimates the coefficients by method-of-moment. Then, *RolyPoly* uses block bootstrap to estimate the variance for the cell type effects, then construct t-statistics to test them (Efron and Tibshirani, 1986). By utilizing GWAS summary statistics for all SNPs near protein-coding genes, the model performed joint analysis with gene expression of a variety of cell types simultaneously, to define prioritized trait-relevant cell types (Calderon et al., 2017). We extracted the log-normalized matrix from each processed data and averaged the expression across each identified cell-type classes. We also scaled the expression data, and then took the absolute expression values, so as to form the input of *RolyPoly* (Calderon et al., 2017). We referred to the Ensembl database (GRCh37) and defined a 10-kb window center around the transcription start site (TSS) of a gene as its transcribed region, to construct a block annotation as recommended that could link the location of GWAS variants with related genes. Of note, we only retained genes on autosomes (Calderon et al., 2017). We used the default parameters and set 1,000 times bootstrap to obtain robust standard errors.

Based on partition heritability, *LDSC-cts* needs the top upregulated genes list of each cell type rather than the expression data (Finucane et al., 2018). Here, we used Wilcoxon rank sum test embedded in *Seurat* to find the DEGs for each cell type with all remaining clusters as control. Following Finucane et al. (2018), we extracted the top 10% upregulated genes ranked by *P* value from each cell type. DEGs were identified as genes expressed in at least 0.1% total cells and with log-transformed fold change above 0 in the target cluster under comparison, so as to ensure a sufficient number of genes could be obtained from each cluster. DEGs lists of each scRNA-seq data used for *LDSC-cts* analysis are summarized in Supplementary Tables 4,8. We referred to the Ensembl database (GRCh37) and defined the region from the TSS to the transcription end sites (TES) of a gene as its transcribed region (Yates et al., 2019). We also added 100-kb windows on either side of the transcribed region of each gene. Finally, we applied *LDSC-cts* by jointly modeling the annotation that corresponded to each cell type, a common annotation that included all of the genes, and the 52 annotations in the default “baseline model,” to identify CLD-specific cell types (Finucane et al., 2018).

We also made a sensitivity analysis. Specifically, we changed the resolution used in clustering to obtain a coarser cell type list for analysis. In particular, since *LDSC-cts* is sensitive to the gene list used for analysis, we simultaneously changed the number of genes included in *LDSC-cts* to the top 5% upregulated ones.

Bonferroni correction was used for multiple tests (*P* < 0.1/n, where *n* = 4 or three is the number of cell type groups, including epithelial cell, non-parenchymal cell, lymphatic immune cell, myeloid immune cell for liver tissue, or gliocyte, neuron, and vascular cell for the brain tissue, Supplementary Table 9) (Hao et al., 2020).

### Statistical Software

We used *scDblFinder* package (version 1.4.0), *Seurat* package (version 1.4.0), *biomaRt* package (version 2.45.6), and *RolyPoly* package (version 0.1.0) in R software (version 3.6.3) (R Core Team, 2020). We used *PLINK* (version 2.0) (Chang et al., 2015) to analyze GWAS data. We also used *LDSC-cts* (version 1.0.1) in python software (version 2.7.18) (Van Rossum and De Boer, 1991).

## Results

### HCC Datasets Analysis

**FIGURE 3:**
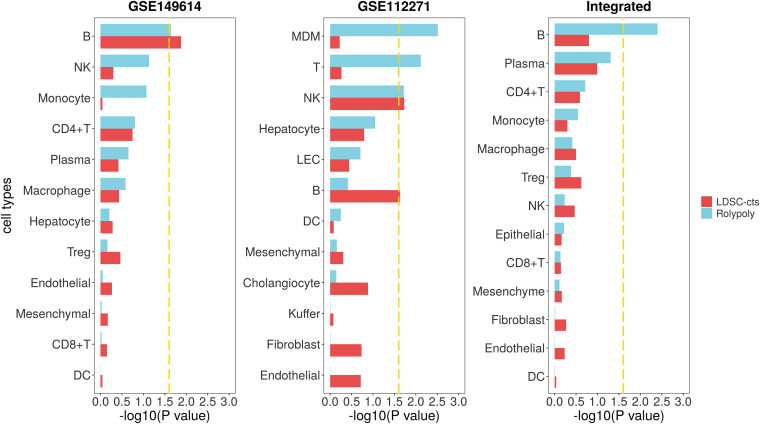
Association between HCC scRNA-seq data and HCC GWAS data from *RolyPoly* and *LDSC-cts.* Using *RolyPoly* and *LDSC-cts* to detect the association of cell types in GSE149614, GSE112271, and their combined HCC scRNA-seq data with EAS HCC GWAS data. Dashed lines in each panel represent a threshold of *P* = 0.1/4. NK, natural killer cell; Treg, regulatory T cell; DC, dendritic cell; MDM, monocyte-derived macrophage; LEC, lymphatic endothelial cell.

For the HCC GWAS data from BBJ, we totally retained 7,246,543 variants with HWE < 10^–6^ and MAF > 0.01, as well as their annotation. For the scRNA-seq data (GSE149614), we identified 14 cell types on 30,983 cells (Supplementary Table 2 and Supplementary Figures 1,2). We further excluded cluster with less than 100 cells (63 mast cells) to avoid the interference of their unstable signal on the results. We also excluded the circulating cluster (2,510 cells), since it usually contains various immune cells from the circulation and may represent a mixed signal. Finally, we retained a total of 28,410 cells from 12 cell types. After integrative analysis, we identified B cell (β = 2.956 × 10^–4^, se = 1.442 × 10^–4^, *P* = 0.0228) as cell type associated with HCC in *RolyPoly* (Figure 3), whereas natural killer cell (NK), monocyte, CD4 + T cell, plasma, macrophage, hepatocyte, regulatory T cell (Treg), endotheliocyte, mesenchymal cell, CD8 + T cell, and dendritic cell (DC) showed no significance (*P* > 0.05). In *LDSC-cts* analysis, we also obtained B cell (β = 2.475 × 10^–9^, se = 1.116 × 10^–9^, *P* = 0.0133) as the significant cell type.

We used another HCC scRNA-seq data from GEO for verification. Totally, we recognized 12 cell types on 30,931 cells from the GSE112271 data with one circulating (1,192 cells) and one small cluster (77 liver sinusoids endothelial cells) excluded (Supplementary Table 2 and Supplementary Figures 3,4). We identified monocyte-derived macrophage (MDM, β = 1.665 × 10^–4^, se = 6.098 × 10^–5^, *P* = 0.0031), T cell (β = 1.732 × 10^–4^, se = 7.170 × 10^–5^, *P* = 0.0076), and natural killer cell (NK, β = 1.458 × 10^–4^, se = 6.976 × 10^–5^, *P* = 0.0191) as cell types significantly associated with HCC in *RolyPoly* (Figure 3), whereas the obtained NK (β = 2.331 × 10^–9^, se = 1.118 × 10^–9^, *P* = 0.0186) and B cell (β = 2.255 × 10^–9^, se = 1.134 × 10^–9^, *P* = 0.0234) as the significant cell types in *LDSC-cts* analysis.

We also integrated the two HCC scRNA-seq data and obtained a combined data consisting of 60,120 cells and 13 cell types for further analysis (Supplementary Figures 5,6). The *RolyPoly* analysis showed that B cell (β = 2.451 × 10^–4^, se = 9.240 × 10^–5^, *P* = 0.0040) was significantly associated with HCC (Figure 3), whereas the *LDSC-cts* identified no significant cell type.

### HCC Dataset Specificity and Sensitivity Analysis

**FIGURE 4:**
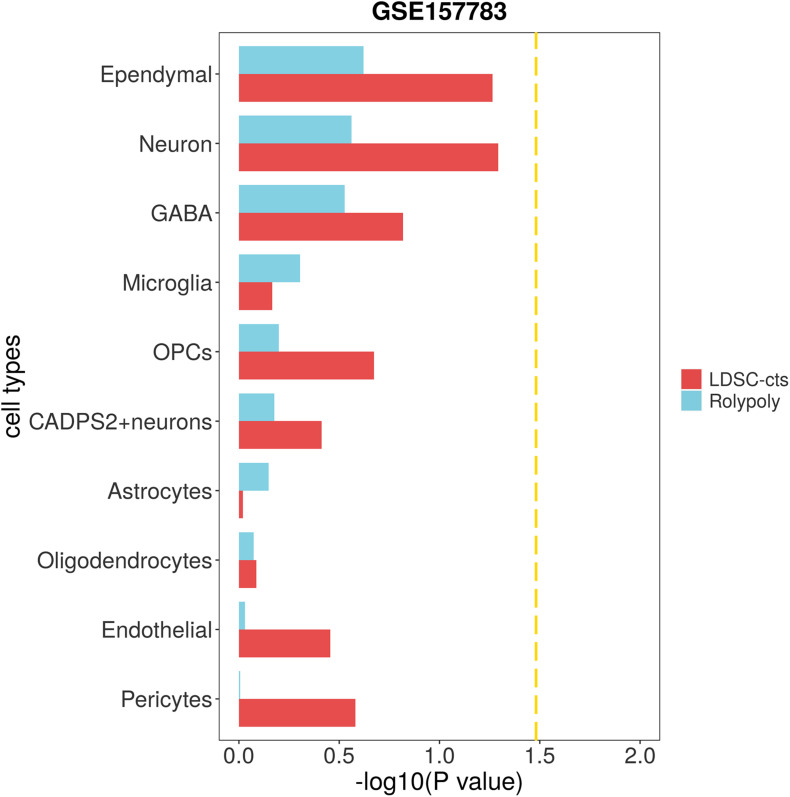
Association of IPD scRNA-seq data with HCC GWAS data form *RolyPoly* and *LDSC-cts*. Using *RolyPoly* and *LDSC-cts* to detect the association of cell types in GSE157783 IPD scRNA-seq data with EAS HCC GWAS data. Dashed lines in each panel represent a threshold of *P* = 0.1/3. OPC, Oligodendrocyte precursor cell; GABA, GABAergic neurons.

We used scRNA-seq data from other disease to verify the specificity of our findings. To be specific, we downloaded one IPD (GSE157783) scRNA-seq data, and identified 12 cell types on 19,002 cells (Supplementary Table 2 and Supplementary Figures 7,8). After excluding clusters with too few cells (47 fibroblasts and 26 T cells), we identified no cell type significantly associated with HCC in either *RolyPoly* or *LDSC-cts* analysis (Figure 4).

We also made a sensitivity analysis by changing the resolution used in clustering and got nine, eight, and nine cell types for GSE149614, GSE112271, and their integrated data, respectively. Sensitivity analysis showed that B cell was still significantly associated with HCC in *RolyPoly* analysis on GSE149614 and the integrated data, as well as in *LDSC-cts* analysis on the integrated data. It also showed nominal significance (*P* < 0.1) in *LDSC-cts* analysis on GSE112271, and was the top cell type (*P* = 0.119) in the analysis on GSE149614 (Supplementary Figure 9).

### Cirrhosis Data Analysis

**FIGURE 5:**
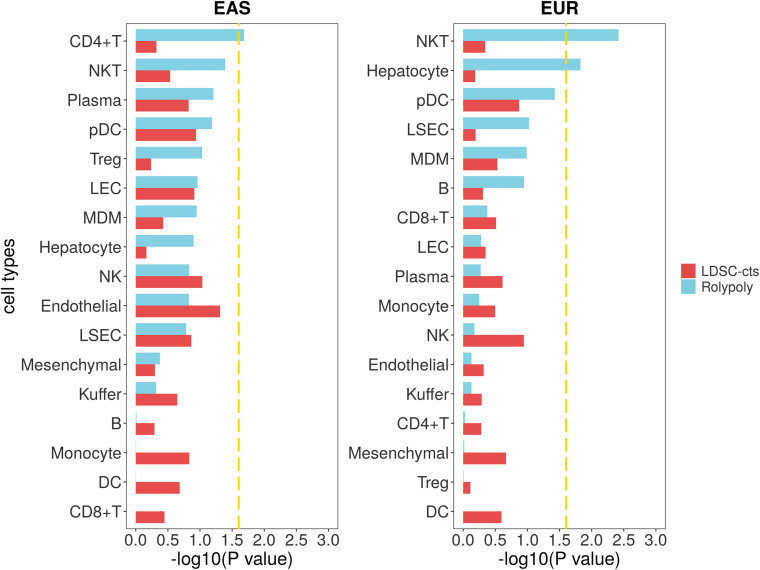
Association between cirrhosis scRNA-seq data and cirrhosis GWAS data from *RolyPoly* and *LDSC-cts*. Using *RolyPoly* and *LDSC-cts* to detect the association of cell types in GSE136103 cirrhosis scRNA-seq data with EAS and EUR cirrhosis GWAS data. Dashed lines in each panel represent a threshold of *P* = 0.1/4. NKT, natural killer T cell; pDC, plasmacytoid dendritic cell; Treg, regulatory T cell; LEC, lymphatic endothelial cell; MDM, monocyte-derived macrophage; NK, natural killer cell; LSEC, liver sinusoids endothelial cell; DC, dendritic cell.

For the cirrhosis GWAS data from BBJ of East Asian population, we totally retained 7,246,475 variants with their annotation. For the scRNA-seq data (GSE136103), we identified 20 cell types on 23,184 cells (Supplementary Table 2; Figure 2; Supplementary Figure 10), but further excluded circulating cluster (309 cells) and clusters with less than 100 cells (56 CLEC9A + dendritic cells and 31 mast cells). Finally, we retained a gene expression data of 17 cell types. *RolyPoly* showed that CD4 + T cell (β = 2.278 × 10^–4^, se = 1.149 × 10^–4^, *P* = 0.0259) was significantly associated with cirrhosis, whereas *LDSC-cts* identified no significant cell type (Figure 5).

We also used a cirrhosis GWAS summary data of European population from GeneATLAS website to verify the stability of our outcomes, in which a total of 7,636,847 variants was retained after QC. We identified natural killer T cell (NKT, β = 6.535 × 10^–10^, se = 2.423 × 10^–10^–1.110 × 10^–9^, *P* = 0.0038) and hepatocyte (β = 2.891 × 10^–10^, se = 1.364 × 10^–10^, *P* = 0.0149) as cell types significantly associated with cirrhosis in *RolyPoly*, while we obtained no significant cell type in the *LDSC-cts* analysis (Figure 5).

## Discussion

Identifying disease-specific cell types has important implications to understand the mechanisms of disease, to guide research, and to develop more precise therapies (Calderon et al., 2017). In this study, using two separate methods and based on available data, we explored the CLD-related cell types through an integrative analysis on GWAS and scRNA-seq data.

In the analysis of HCC, both *RolyPoly* and *LDSC-cts* identified B cell as significant associated with HCC (*P* = 0.0228 and *P* = 0.0133, respectively). B cell mainly exerts its humoral immunity function through the antibody production and antigen presentation, and can also regulate T cells and innate immune responses (Tsou et al., 2016). Recently, the regulation role of resident B cell in tumor has been investigated (Garaud et al., 2018; Lechner et al., 2019; Wang et al., 2019). The balance between B cells in different states and their activities may have the potential to affect pro- or anti-tumor functions (Largeot et al., 2019; Liu et al., 2019). A similar phenomenon has also been observed in liver disease. In a Hras12V HCC mouse models, B cells were found to have a potential role in suppressing hepatic tumorigenesis (Wang et al., 2017), whereas in another mouse model with inflammation-associated HCC, infiltrating B cells was correlated with increased tumor aggressiveness and mortality (Faggioli et al., 2018). In addition, activated FcγRII^*low/*–^ B cells from HCC tumor may also suppress host anti-tumor immune response via IL-10 signals (Ouyang et al., 2016; Jin et al., 2017). Nevertheless, the depth of research on tumor-associated B cells and their subsets is far less than that of T cells. As for the liver diseases, existing several unbiased scRNAseq research on CLD have not revealed major alterations in the composition or transcriptional profile of liver B cells in disease state (MacParland et al., 2018; Ramachandran et al., 2019; Losic et al., 2020; Sharma et al., 2020). Separate single-cell research has not been conducted specifically on the relationship between B cells and liver disease. However, with the development of single-cell technology, the combination of single-cell transcriptomics and immunomics (B cell receptor) is expected to further reveal the exact role of B cells in HCC and other CLD, and explore B cell-based immunotherapy (Setliff et al., 2019).

We also used another HCC-related scRNA-seq data to verify our findings. *RolyPoly* identified MDM, T cell, and NK cell, rather than B cell, as significant cell types, whereas B cell remained significant together with NK cell in *LDSC-cts* analysis. This might have resulted from *LDSC-cts* using DEGs, which may be conserved but more robust among different studies for a specific disease. Although we have averaged the expression for each identified cell type and taken a scale on the averaged data, differences in data structure arising from the different angles of the two original studies may also be a probable interpretation (Losic et al., 2020). Therefore, we further integrated the two data and repeated these analyses, and found that B cell regained its significance in the integrated data under *RolyPoly* method. In addition, we used the IPD scRNA-seq data (GSE157783) from brain tissue to make specificity analysis, and found that neither *RolyPoly* nor *LDSC-cts* method identified significant cell types. The above results jointly indicated that B cells may be a significant cell type for HCC, and more attention should be paid to them in future research.

Of note, outcomes from the second HCC data also suggested that NK cells might be HCC-related cells, which was significant in both *RolyPoly* and *LDSC-cts* analysis. Although this result has not been verified in our analysis, a previous study has identified the contribution of NK cell in liver injury (Luci et al., 2019), NK cell composition alteration and an interaction with other clusters was also observed in HCC (Zhang et al., 2019). Thus, it is also of meaning to further explore the relationship between NK cell and HCC.

As for the analysis on cirrhosis, we have not obtained an overlap cell type in the two methods, with CD4 + T cell significant in *RolyPoly* analysis using the GWAS data on East Asian population, while NKT and hepatocyte are significant in *RolyPoly* analysis on European population. That might be caused by the different linkage disequilibrium and minor allele frequency (MAF) for different ancestry, cross-population correlations of causal SNP effects, and heritability (Mather and Thalamuthu, 2020; Wang et al., 2020; Yang and Zhou, 2020). For example, there are 1,558 SNPs and 76 SNPs with *P* < 10^–6^ in EAS and EUR datasets, respectively (Supplementary Table 10).

Certainly, several limitations remain in our study. First, all data used came from public databases, and external experiments were not conducted to verify our findings; but alternatively, we used other available GWAS and scRNA-seq data to make verification as well as specificity analysis, which would also ensure the reliability of our results to some extent. Second, *SCTransform* is a relative powerful normalization method, which may weaken the heterogeneity among samples when used for integration (Butler et al., 2018; Tran et al., 2020). Since we were aimed to apply similar cell type definition strategy in different samples and focused mainly on the similarity rather than heterogeneity, it may offer more help than interference to our analysis. In addition, since current research advances have limited ability in cell type definition and explanation, we applied a relative conservation cell subdivided strategy in the current study. With the in-depth research on various cell subtypes and the development of single-cell technology, similar research is expected be carried out in a larger sample with a higher resolution and precision, and more novel findings with biological explanation would be obtained.

In summary, we performed integrative analysis on GWAS summary data and single scRNA-seq data of CLD, and identified B cell as a potential HCC-related cell type. Since we have made verification from multiple angles, our outcomes are of relative reliability. In addition, as the single-cell atlas of different tissues and diseases has been completed, more targeted researches are expected, and our study would provide valuable clues for further research on CLD.

## Code Availability

Code used for data processing, integrated analysis, and plotting could be found at (https://github.com/XiangyuYe/CLD-specific-celltype-identification).

## Data Availability Statement

Asian ancestry CLD GWAS summary data was downloaded from BBJ (http://jenger.riken.jp/en/). European ancestry CLD GWAS summary data was downloaded from GeneATLAS website (http://geneatlas.roslin.ed.ac.uk/). ScRNA-seq datasets used (GSE136103, GSE149614, GSE112271, and GSE157783) were downloaded from the GEO database (https://www.ncbi.nlm.nih.gov/geo/).

## Author Contributions

RBY and PH designed the study. JLW, YFW, and MLZ performed the datasets quality control. XYY and YW performed the data analysis. PH, HBC, and YFZ interpreted the analysis results. XYY and JLW wrote the draft manuscript. RBY, PH, and MY revised the article. All authors accepted the final manuscript.

## Conflict of Interest

The authors declare that the research was conducted in the absence of any commercial or financial relationships that could be construed as a potential conflict of interest.

## Footnotes

1 http://jenger.riken.jp/en/

2 http://geneatlas.roslin.ed.ac.uk/
