# Supplementary material for: Leveraging Single-Cell RNA-seq Data to Uncover the Association Between Cell Type and Chronic Liver Diseases
Source: Front Genet. 2021 Mar 8;12:637322. doi: 10.3389/fgene.2021.637322 (PMC7982650; doi:10.3389/fgene.2021.637322)
Supplement: Supplementary file 1 [file Data_Sheet_1.PDF]

Supplementary figures:

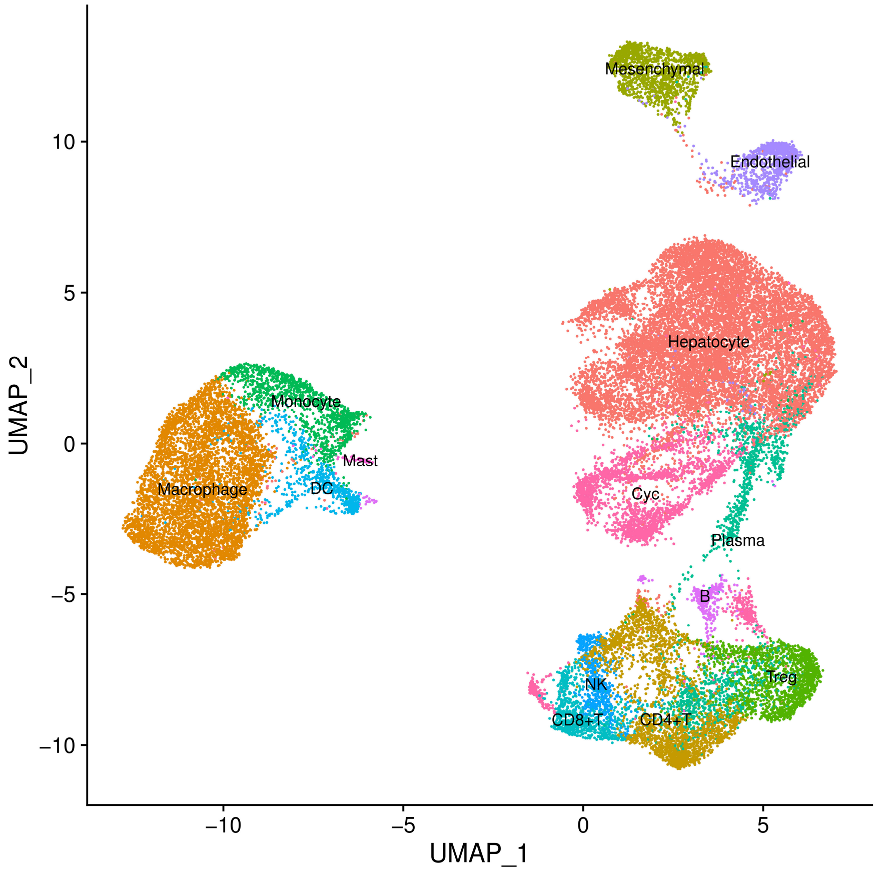

Figure S1 Cell types inferred from expression of marker gene signatures in GSE149614.

Abbreviation: Treg: regulatory T cell, DC: dendritic cell, NK: natural killer cell

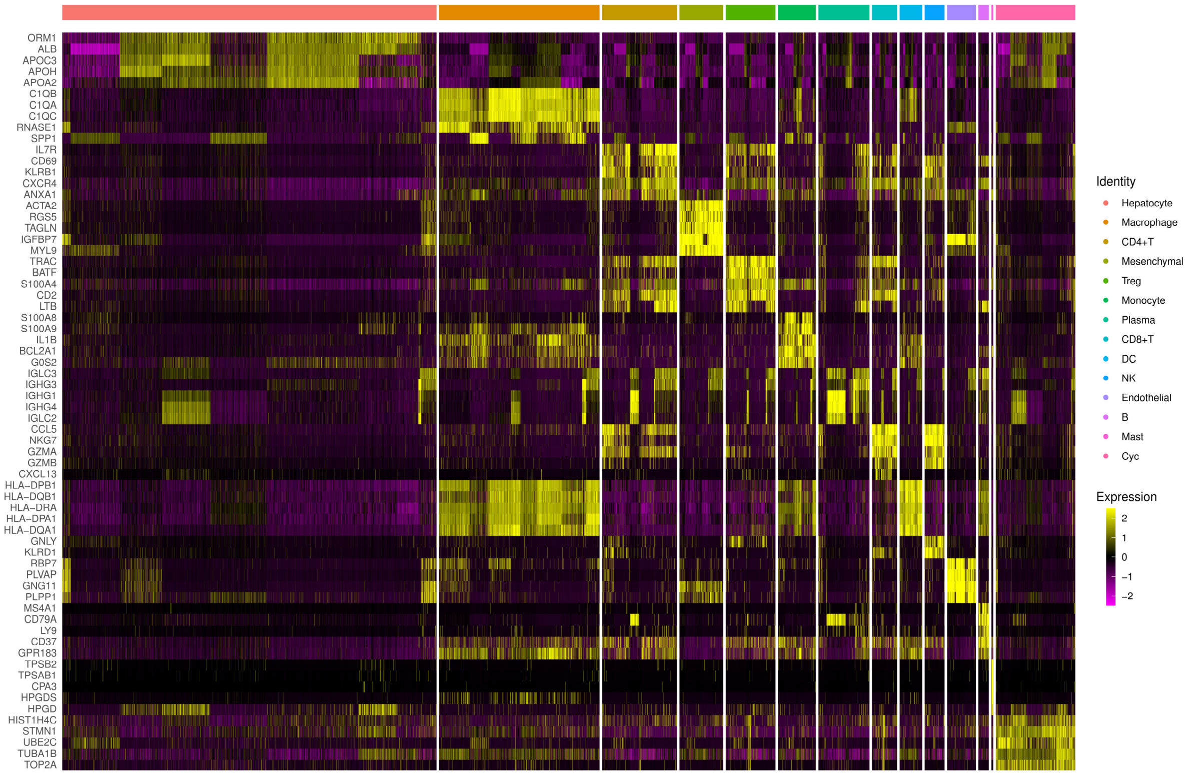

Figure S2 Heat map of cluster marker genes in GSE149614

Columns denote cells; rows denote genes. Abbreviation: Treg: regulatory T cell, DC: dendritic cell, NK: natural killer cell

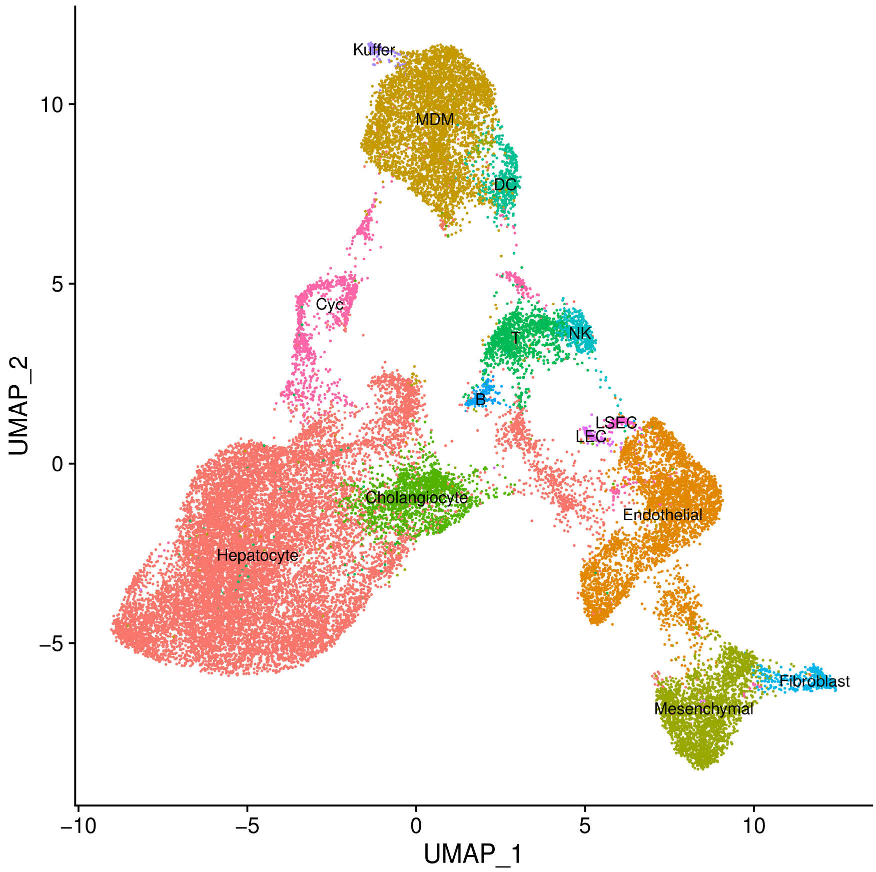

Figure S3 Cell types inferred from expression of marker gene signatures in GSE112271.

Abbreviation: MDM: monocyte-derived macrophage, DC: dendritic cell, NK: natural killer cell, LEC: lymphatic endothelial cell, LSEC: liver sinusoids endothelial cell

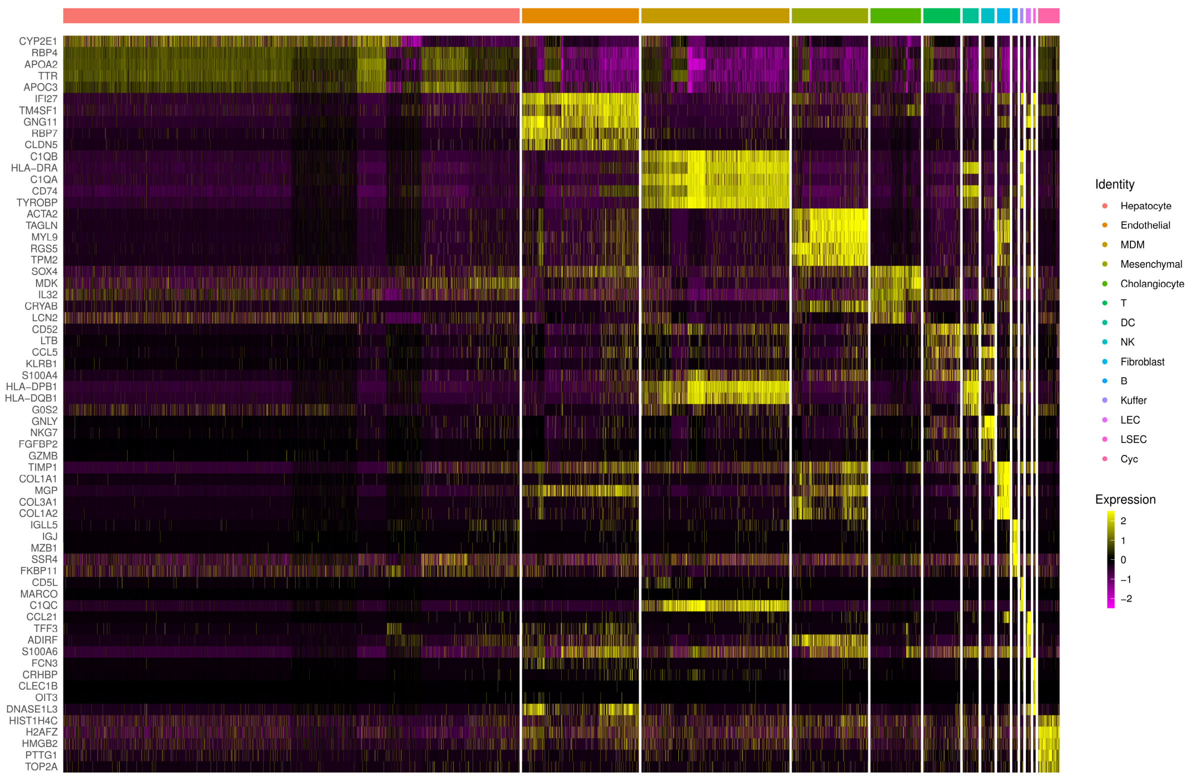

Figure S4 Heat map of cluster marker genes in GSE112271

Columns denote cells; rows denote genes. Abbreviation: MDM: monocyte-derived macrophage, DC: dendritic cell, NK: natural killer cell, LEC: lymphatic endothelial cell, LSEC: liver sinusoids endothelial cell

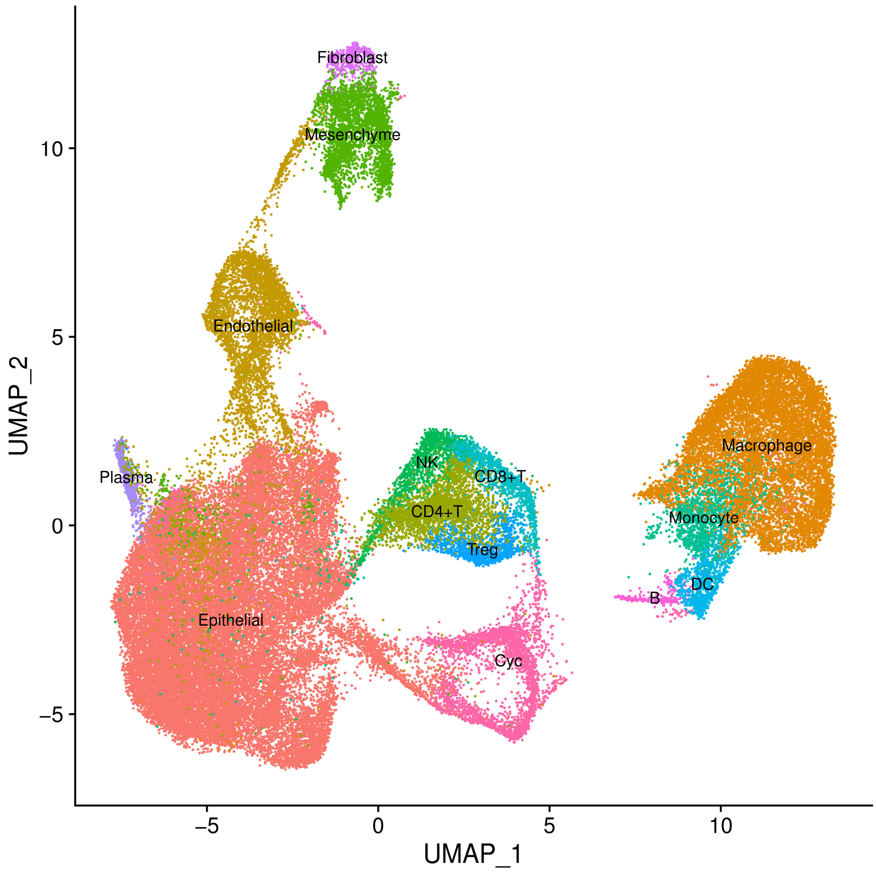

Figure S5 Cell types inferred from expression of marker gene signatures in integrated data (GSE149614 and GSE112271)

Abbreviation: NK: natural killer cell, DC: dendritic cell, Treg: regulatory T cell

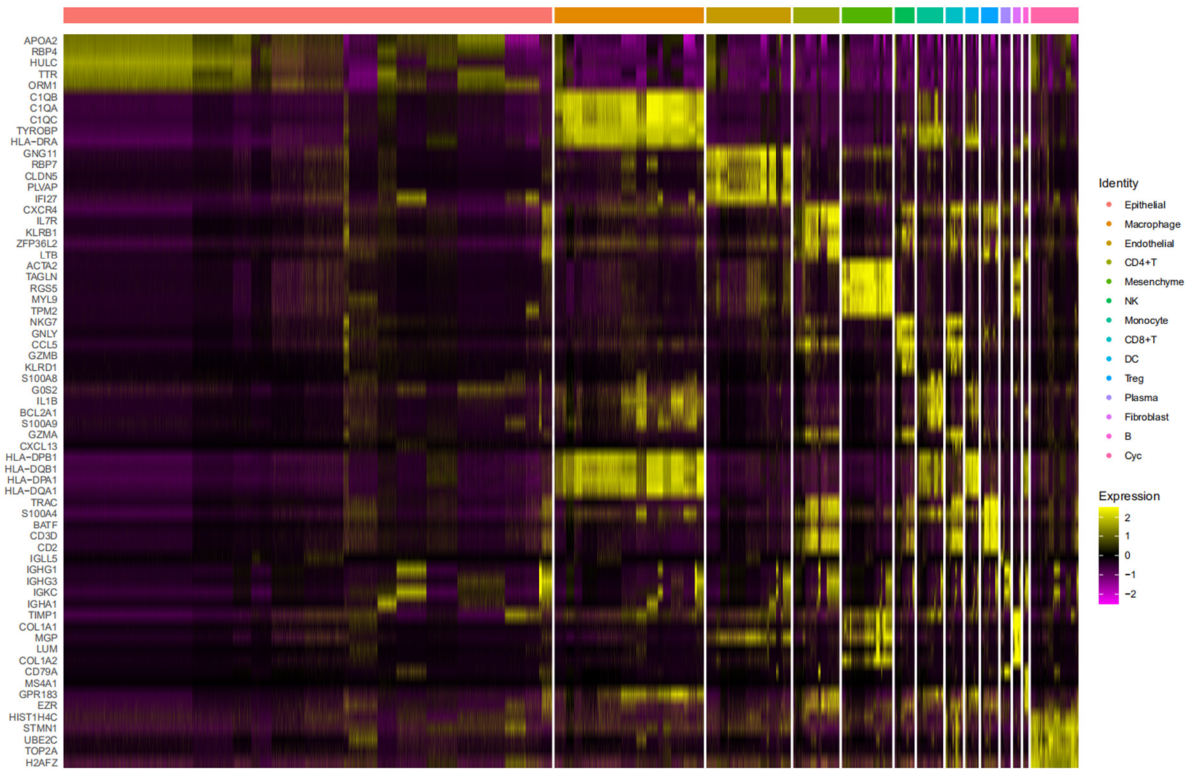

Figure S6 Heat map of cluster marker genes in integrated data (GSE149614 and GSE112271)

Columns denote cells; rows denote genes. Abbreviation: NK: natural killer cell, DC: dendritic cell, Treg: regulatory T cell

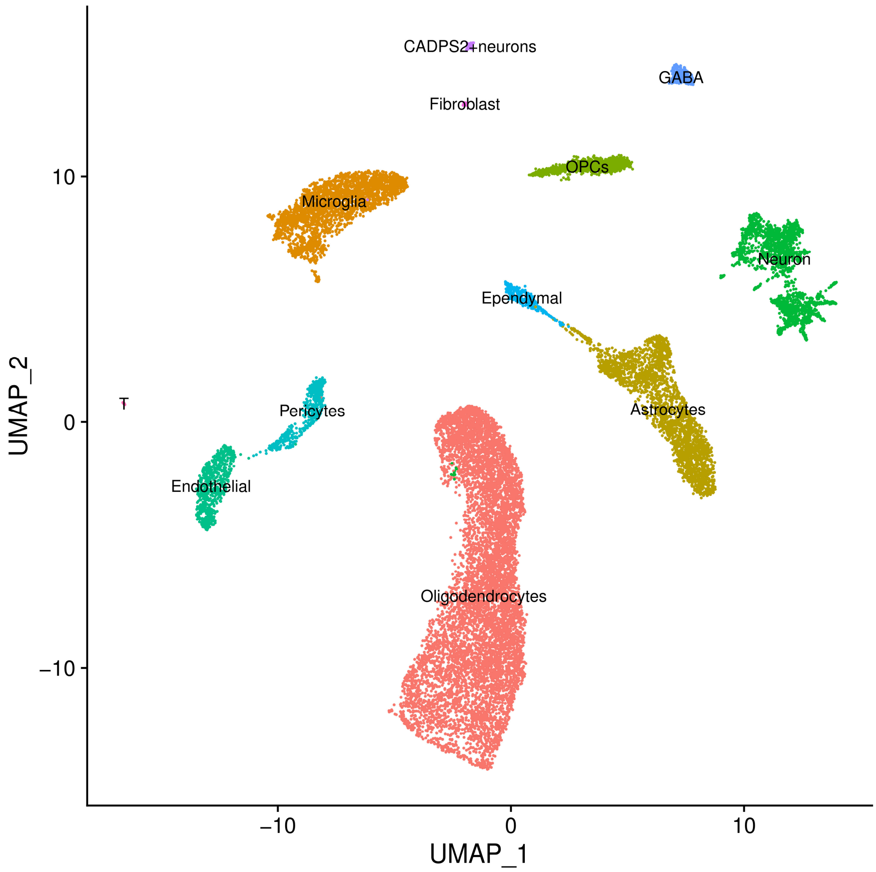

Figure S7 Cell types inferred from expression of marker gene signatures in GSE157783

Abbreviation: OPC: Oligodendrocyte precursor cell, GABA: GABAergic neurons

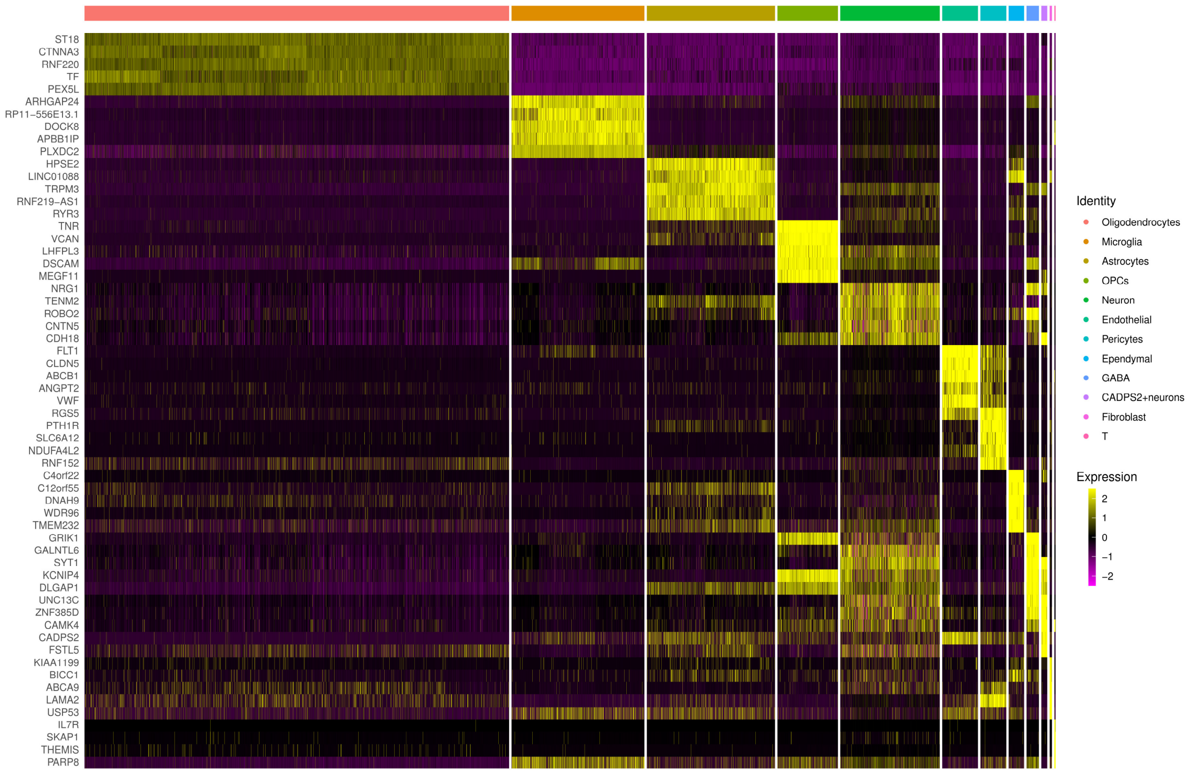

Figure S8 Heat map of cluster marker genes in GSE157783

Columns denote cells; rows denote genes. Abbreviation: OPC: Oligodendrocyte precursor cell, GABA: GABAergic neurons

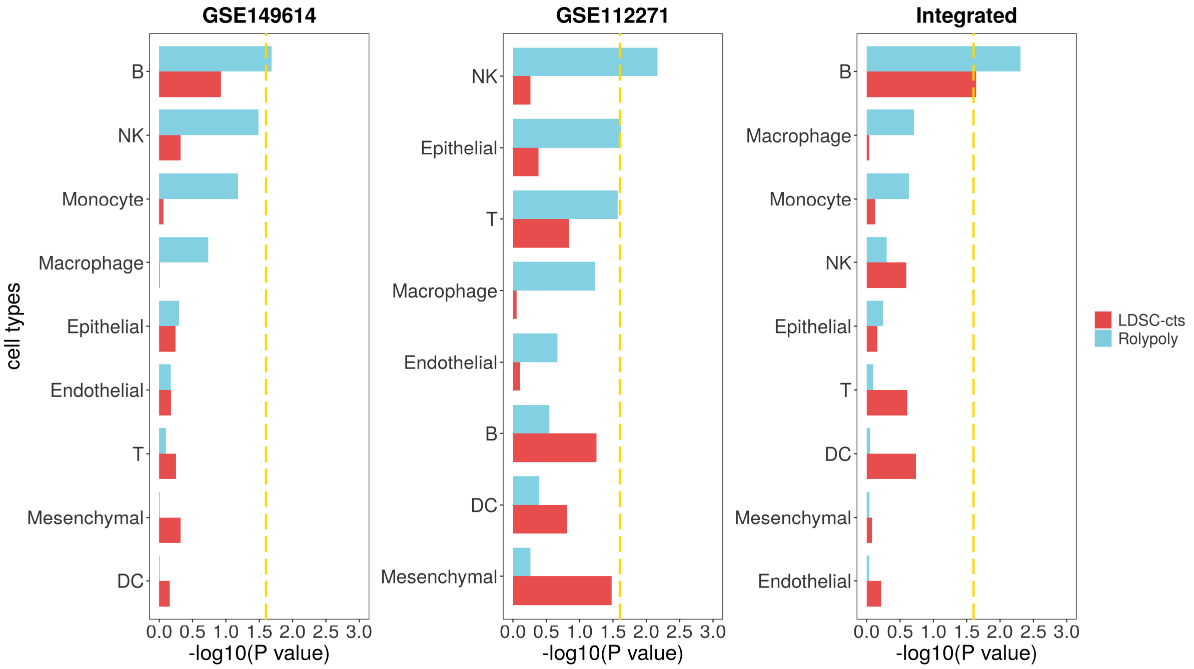

Figure S9 Sensitivity analysis on the association between HCC scRNA-seq data and HCC GWAS data from *RolyPoly* and *LDSC-cts*

Using Rolypoly and LDSC-cts to detect the association of cell types in GSE149614, GSE112271 and their integrated HCC scRNA-seq data with EAS HCC GWAS data after changing the resolution used for clustering (and number of genes for *LDSC-cts*). Dashed lines in each panel represent a threshold of *P*=0.1/4. Abbreviation: NK: natural killer cell, Treg: regulatory T cell, DC: dendritic cell, MDM: monocyte-derived macrophage, LEC: lymphatic endothelial cell

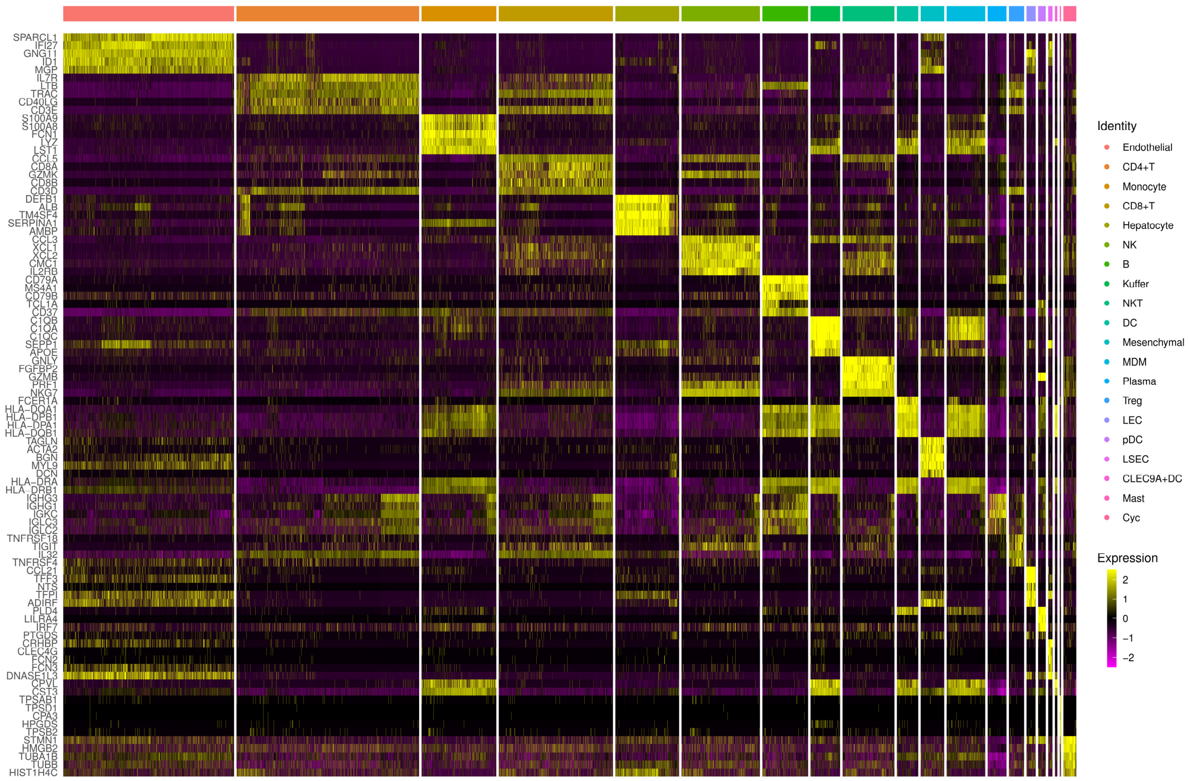

Figure S10 Heat map of cluster marker genes in GSE136103

Columns denote cells; rows denote genes. Abbreviation: NKT: natural killer T cells, pDC: plasmacytoid dendritic cell, Treg: regulatory T cell, LEC: lymphatic endothelial cell, MDM: monocyte-derived macrophage, NK: natural killer cell, LSEC: liver sinusoids endothelial cell, DC: dendritic cell
